# Nutrigenetic Interaction of Spontaneously Hypertensive Rat Chromosome 20 Segment and High-Sucrose Diet Sensitizes to Metabolic Syndrome

**DOI:** 10.3390/nu14163428

**Published:** 2022-08-20

**Authors:** Ondřej Šeda, Kristýna Junková, Hana Malinska, Adéla Kábelová, Martina Hüttl, Michaela Krupková, Irena Markova, František Liška, Lucie Šedová

**Affiliations:** 1 Institute of Biology and Medical Genetics, The First Faculty of Medicine, Charles University and the General University Hospital in Prague, 128 00 Prague, Czech Republic; 2 Centre for Experimental Medicine, Institute for Clinical and Experimental Medicine, 140 21 Prague, Czech Republic

**Keywords:** metabolic syndrome, congenic rat, animal model, nutrigenetics

## Abstract

Several corresponding regions of human and mammalian genomes have been shown to affect sensitivity to the manifestation of metabolic syndrome via nutrigenetic interactions. In this study, we assessed the effect of sucrose administration in a newly established congenic strain BN.SHR20, in which a limited segment of rat chromosome 20 from a metabolic syndrome model, spontaneously hypertensive rat (SHR), was introgressed into Brown Norway (BN) genomic background. We mapped the extent of the differential segment and compared the genomic sequences of BN vs. SHR within the segment in silico. The differential segment of SHR origin in BN.SHR20 spans about 9 Mb of the telomeric portion of the short arm of chromosome 20. We identified non-synonymous mutations e.g., in *ApoM*, *Notch4*, *Slc39a7*, *Smim29* genes and other variations in or near genes associated with metabolic syndrome in human genome-wide association studies. Male rats of BN and BN.SHR20 strains were fed a standard diet for 18 weeks (control groups) or 16 weeks of standard diet followed by 14 days of high-sucrose diet (HSD). We assessed the morphometric and metabolic profiles of all groups. Adiposity significantly increased only in BN.SHR20 after HSD. Fasting glycemia and the glucose levels during the oral glucose tolerance test were higher in BN.SHR20 than in BN groups, while insulin levels were comparable. The fasting levels of triacylglycerols were the highest in sucrose-fed BN.SHR20, both compared to the sucrose-fed BN and the control BN.SHR20. The non-esterified fatty acids and total cholesterol concentrations were higher in BN.SHR20 compared to their respective BN groups, and the HSD elicited an increase in non-esterified fatty acids only in BN.SHR20. In a new genetically defined model, we have isolated a limited genomic region involved in nutrigenetic sensitization to sucrose-induced metabolic disturbances.

## 1. Introduction

Metabolic syndrome (MetS) comprises a cluster of multifactorial conditions, including insulin resistance, dyslipidemia, central obesity, and hypertension [1]. Its prevalence is on the rise worldwide [2]. MetS presents a significant health burden both individually and on the societal level; therefore deciphering its architecture is crucial for devising effective predictive, preventive, and therapeutic modalities. While it is acknowledged that complex interactions between environmental and genomic components are essential for the pathogenesis of metabolic syndrome and all of its individual features [3], their detailed analysis is complicated by many hindrances. Model systems allow for standardizing and defining the main factors, e.g., for nutrigenetic interactions, diets of defined composition can be fed to the genetically designed animal strains differing only in the selected genomic regions or gene variants. In comparative and translational research of cardiovascular and metabolic conditions, the rat *(Rattus norvegicus*) has been a model of choice for decades [4]. With the availability of complete genome sequences of many different inbred rat strain models, this suitability extended to the analyses of the genome-environmental architecture of complex conditions [5,6]. We and others have repeatedly shown that variations in several rat (and their corresponding human) genomic regions affect simultaneously several or all components of the metabolic syndrome, either through pleiotropy or clustering of independent actions of relevant genes. In particular, regions of rat chromosomes (RNO) 1 [7], 2 [8,9], 4 [10,11], 8 [12], 16 [13], 17 [14,15], and 20 [16,17] were studied in this respect. Transferring the respective chromosomal segments between strains resulted in the manifestation or amelioration of metabolic syndrome in the created congenic strains. While several regions mentioned above seem to affect metabolic syndrome-related phenotypes constitutively, loci on RNO 4, 8, and 20 were reported to participate in nutrigenetic [9,16,18] or pharmacogenetic [18,19,20] interactions modulating the sensitivity towards the metabolic syndrome.

One of the most exhaustively studied models of metabolic syndrome is the Spontaneously Hypertensive Rat (SHR). In the case of RNO20, the genomic region of SHR origin encompassing the major histocompatibility complex (Rt1) was associated with blood pressure in a set of recombinant inbred rat strains already in 1989 [21]. Indeed, the derived congenic strain SHR.BN-RT1^n^ (SHR.1N) carrying the RNO20 differential segment, including Rt1 of Brown Norway origin on SHR background, showed lower blood pressure and a less favorable lipid profile [21,22]. However, subsequently it was revealed that, when exposed to a high-calorie diet, the SHR.1N displays greater weight gain, increased adiposity, and worse glucose tolerance than similarly challenged SHR [17,23]. In this study, we aimed to dissect the potential nutrigenetic interaction(s) in a novel, complementary genetic model system, where SHR RNO20 segment is introduced into the Brown Norway genomic background.

## 2. Materials and Methods

### 2.1. Ethical Statement

All experiments were performed in agreement with the Animal Protection Law of the Czech Republic. The experimental protocols and detailed procedures were evaluated and approved by the Ethical Committee of the First Faculty of Medicine, Charles University in Prague, and by the Ministry of Education, Youth and Sports of the Czech Republic (permit 8615/2019-MZE-17214). The health of the rats was examined daily, and the animals were monitored every hour during the experimental procedures. There were no unexpected deaths during the experiments.

### 2.2. Derivation of the BN.SHR20 Congenic Rat Strain

The SHR/OlaIpcv [SHR hereafter, Rat Genome Database (RGD) [24], ID no. 631848] and BN/Cub (RGD ID no. 737899) strains were maintained at the Institute of Medical Biology and Genetics, Charles University in Prague. To derive the BN.SHR20 congenic strain, we used a marker-assisted backcross breeding approach, as described previously [13,16,25]. In short, SHR rats were crossed with BN/Cub rats, and the subsequent F1 hybrids were repeatedly backcrossed to BN/Cub. The differential segment was fixed by intercrossing heterozygotes and selecting the progeny with homozygous SHR-derived chromosome 20 segments. The congenic status of the new BN.SHR20 strain was validated with a whole-genome marker scan.

### 2.3. DNA Extraction and Genotyping

Rat DNA was isolated from tail samples by the modified phenol extraction method. Primer nucleotide sequences were obtained from public databases (via RGD). Polymerase chain reaction (PCR) was used for genotyping markers polymorphic between progenitor strains. We tested DNA from the congenic strain (BN.SHR20, n = 20) and the progenitor strains SHR and BN/Cub. The PCR products were separated on polyacrylamide (7–10%) gels and detected in UV light after ethidium bromide staining using Syngene G:Box (Synoptics, Ltd., Cambridge, UK).

### 2.4. Experimental Protocol

Adult male rats were housed under temperature—(23 °C) and humidity—(55%) controlled conditions on 12-h light/12-h dark cycle and fed a laboratory chow diet (STD, ssniff RZ, ssniff Spezialdiäten GmbH, Soest, Germany). Animals had free access to food (standard chow) and water at all times. At 4 months of age, males from the BN.SHR20 congenic strain (n = 20) and the parental BN/Cub strain (n = 16) were randomly assigned to control and experimental groups. The control groups continued to be fed STD, while the experimental groups were fed high-sucrose diet (HSD, protein (19.6 cal%), fat (10.4 cal%), carbohydrates (sucrose, 70 cal%) prepared by Institute for Clinical and Experimental Medicine, Prague, Czech Republic, described previously in detail [26]), for 14 days. At the end of the experiment, all rats were subjected to an oral glucose tolerance test (OGTT) after overnight fasting, and blood samples were drawn for further biochemical analyses. The animals were then sacrificed, and their total weight and the weights of the heart, liver, kidneys, and the epididymal and retroperitoneal fat pads, were determined.

### 2.5. Metabolic Measurements

The OGTT was performed after overnight fasting. Blood samples for glycemic assessment (Ascensia Elite Blood Glucose Meter, Bayer HealthCare, Mishawaka, IN, USA) were obtained from the tail vein at intervals of 0, 30, 60, 120, and 180 min after intragastric glucose administration to conscious rats (3 g/kg body weight, 30% aqueous solution). Serum triacylglycerol (TG) and cholesterol concentrations were measured by standard enzymatic methods (Erba-Lachema, Brno, Czech Republic). Serum non-esterified fatty acids (NEFA) were measured with an acyl-CoA oxidase-based colorimetric kit (Roche Diagnostics GmbH, Mannheim, Germany). Enzyme-linked immunosorbent assay (ELISA) kits were used to determine the serum levels of insulin (Mercodia, Uppsala, Sweden).

### 2.6. In Silico Analyses

To compare the publicly available DNA sequences of SHR and BN rat strains, we used the Variant Visualizer resource provided by the RGD at http://rgd.mcw.edu/ (accessed on 7 July 2022) with high conservation settings (0.75–1) determined by PHAST [25], minimum read depth set to eight, and exclusion of variants found in fewer than 15% of reads. The results were then verified in the relevant NCBI-based databases. The Virtual Comparative Map software tool at http://www.animalgenome.org/VCmap (accessed on 7 July 2022) and the Gene and Ortholog Location Finder (GOLF) provided by the RGD were used to identify the regions of the human genome syntenic to the differential segment in the BN.SHR20 congenic strain. These regions were then examined for the presence of the significant associations (SNP-trait associations with *p*-value ≤ 5.0 × 10^−8^) reported in human genome-wide association studies (extracted from the Catalog of Published Genome-Wide Association Studies, available at: http://www.ebi.ac.uk/gwas, 7 July 2022, [27]).

### 2.7. Statistical Analysis

All statistical analyses were performed in STATISTICA, version 14.0 (TIBCO Software Inc., Palo Alto, CA, USA). When comparing morphometric and biochemical variables between groups, two-way ANOVA with STRAIN and DIET as major factors were used, followed by post-hoc Tukey’s test for pairwise comparisons. Null hypothesis was rejected whenever *p* > 0.05.

## 3. Results

### 3.1. Genomic Characterization of the BN.SHR20 Congenic Strain

**Figure 1:**
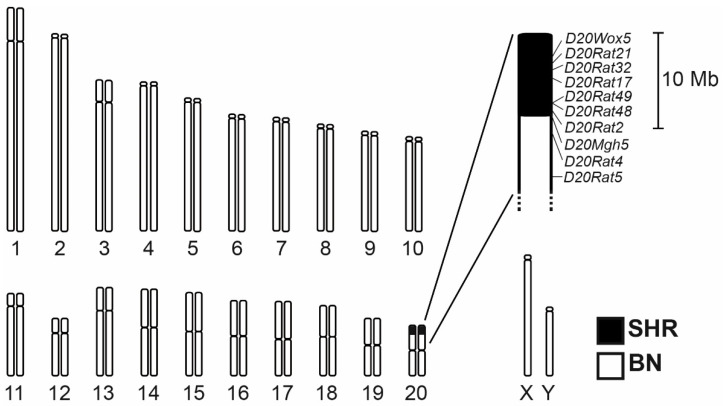
The chromosome 20 differential segment in the BN.SHR20 congenic strain. The SHR-derived region is shown in black. The detailed view shows the markers that were genotyped in determining the extent of the differential segment. Their position is shown according to the *Rattus norvegicus* mRatBN7.2 genome assembly.

The genotyping scan including a set of 34 markers polymorphic between SHR and BN on rat chromosome 20 (RNO20) revealed the extent of the differential segment in the BN.SHR20 (tel-D20Mhg5)/Cub congenic strain (BN.SHR20 hereafter). The differential segment spans about 9 Mb of the telomeric portion of the RNO20 short arm (Figure 1). Several total genome scans were conducted during the derivation of the BN.SHR20 strain, excluding the presence of non-SHR alleles other than those fixed on RNO20, confirming the congenic status of the new strain. The SHR-derived RNO20 segment hence represents the only genomic difference between BN and BN.SHR20 strains.

### 3.2. Nutrigenetic Effects of the RNO20 Differential Segment

**Figure 2:**
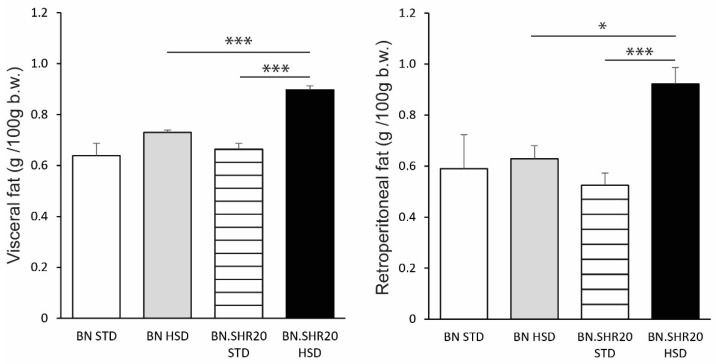
Adipose tissue depots in BN and BN.SHR20 rats. The relative weights of visceral (left) and retroperitoneal (right) adipose tissue depots in sucrose-fed (HSD) and control (STD) adult male rats of BN vs. BN.SHR20 strains. Within the graphs, the significance levels of pairwise comparisons by post-hoc Tukey’s honest significance difference test of the two-way ANOVA with STRAIN and DIET as major factors are indicated as follows: * *p* < 0.05; *** *p* < 0.001.

Body weight did not differ between strains in control or HSD-fed groups. While HSD led to an increase in body weight in BN (BN-STD: 209 ± 9 g vs. BN-HSD: 238 ± 3 g, *p* = 0.025), there was no effect on the relative weights of either visceral or retroperitoneal adipose tissue depots in this strain (Figure 2). On the contrary, BN.SHR20 showed a marked increase in adiposity (Figure 2) despite no significant change in the total body weight (BN.SHR20-STD: 226 ± 3 g vs. BN.SHR20-HSD: 241 ± 8 g, *p* = 0.08).

**Figure 3:**
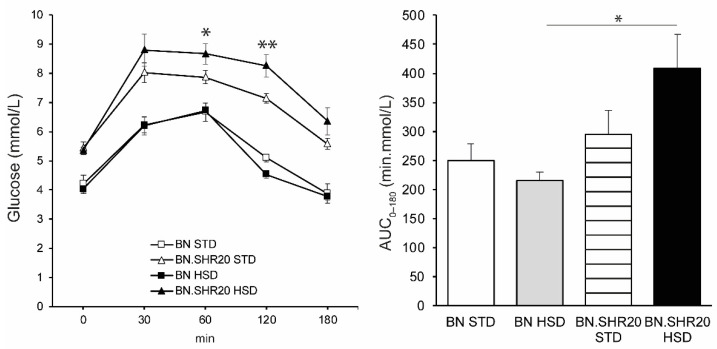
Oral glucose tolerance test and the area under the glycemic curve (AUC_0–180_). The course of glycemia during OGTT is shown (left) in sucrose-fed (HSD, closed symbols) and control (STD, open symbols) adult male rats of BN (squares) vs. BN.SHR20 (triangles) strains. Within the graphs, the significance levels of pairwise comparisons (BN.SHR20 STD vs. HSD for OGTT; BN-HSD vs. BN.SHR20-HSD for AUC) by post-hoc Tukey’s honest significance difference test of the two-way ANOVA with STRAIN and DIET as major factors are indicated as follows: * *p* < 0.05; ** *p* < 0.01.

**Figure 4:**
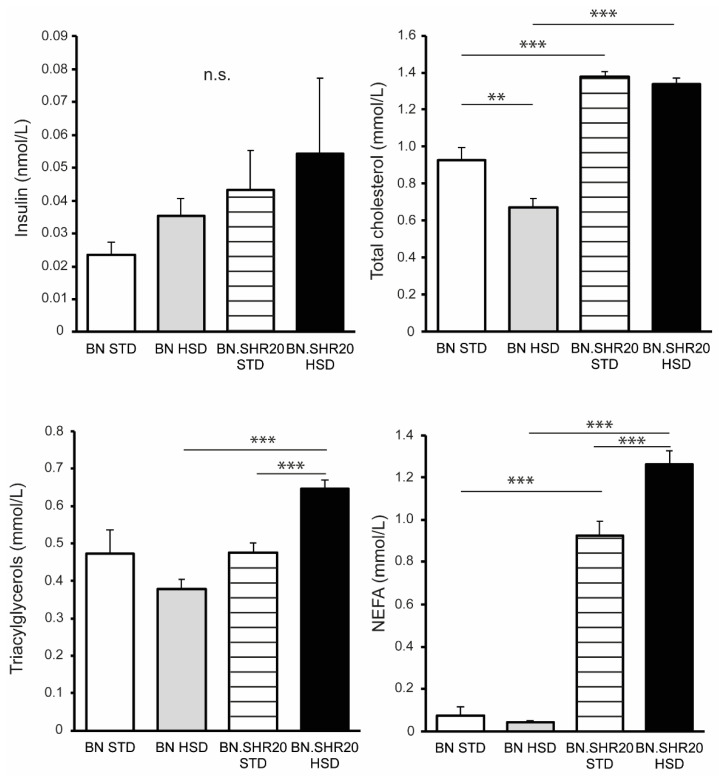
Metabolic profile of BN and BN.SHR20 rats. The levels of fasting insulin, total cholesterol, triacylglycerols, and non-esterified fatty acids (NEFA) in sucrose-fed (HSD) and control (STD) adult male rats of BN vs. BN.SHR20 strains. Within the graphs, the significance levels of pairwise comparisons by post-hoc Tukey’s honest significance difference test of the two-way ANOVA with STRAIN and DIET as major factors are indicated as follows: n.s.: not significant; ** *p* < 0.01; *** *p* < 0.001.

Fasting glycemia and the glucose levels during the entire oral glucose tolerance test showed a significant effect of STRAIN factor in two-way ANOVA (Supplementary Table S1) as all BN.SHR20 values were higher compared to BN (Figure 3). Furthermore, in BN.SHR20 only, the glucose concentrations at 60th and 120th minutes of the test were elevated by HSD. This resulted in a higher residual area under the glycemic curve in HSD-fed BN.SHR20 vs. HSD-fed BN rats (Figure 3), while fasting concentration of insulin did not differ significantly among groups (Figure 4).

We identified significant STRAIN × DIET interactions for the non-esterified fatty acids, triacylglycerols, and total cholesterol (Supplementary Table S1). The non-esterified fatty acids and total cholesterol concentrations were higher in BN.SHR20 compared to their respective BN groups, and the HSD elicited an increase in non-esterified fatty acids only in BN.SHR20 (Figure 4). HSD-fed BN rats showed slightly lower total cholesterol in comparison with their STD-fed controls. The fasting levels of triacylglycerols were the highest in sucrose-fed BN.SHR20, both compared to the sucrose-fed BN and the control BN.SHR20 (Figure 4).

### 3.3. Prioritization of Candidate Genes

The differential segment of the BN.SHR20 congenic strain harbors 627 annotated genes (NCBI Rattus norvegicus Annotation Release 108, Rattus norvegicus mRatBN7.2 (GCF_015227675.2 assembly)), including the complete major histocompatibility (Rt) system. We compared the genomic DNA sequences throughout the BN.SHR20 differential segment between the two parental strains in silico to identify highly conserved variations. In this manner, we identified a total of 3932 differences between SHR and BN strains, both within genes (n = 193) and the intergenic regions (Supplementary Table S2). Among these, there were 48 protein-coding genes within the segment that were predicted to carry exonic mutations (both synonymous and non-synonymous), most of them pertaining to the major histocompatibility complex-Rt1 (Supplementary Table S3). As it is clear that physiologically relevant changes may arise both from within and outside of the coding regions, we compared the SHR vs. BN sequence variations with syntenic sections of the human genome with reported highly significant associations in human genome-wide studies. There were 54 cases when human single nucleotide polymorphisms showed an association to one or more metabolic syndrome components and, at the same time, there was a genomic variation between SHR and BN in the syntenic locus (Table 1).

## 4. Discussion

The metabolic syndrome and its components arise as a result of a higher-order network of interactions between environmental, genomic, epigenomic and metagenomic factors [28,29]. While hundreds of DNA variations have been associated with MetS or its individual features [30] and the detrimental role of high-calorie, particularly fructose-based diets in the pathogenesis of MetS has been firmly established [31,32], there is still only limited information concerning the involved nutrigenetic interactions. Here, we isolated a narrow genomic region of rat chromosome 20 sensitizing to the effects of high-sucrose diets on adiposity, dyslipidemia, and glucose tolerance. Congenic BN.SHR20 rats responded to sucrose feeding with a deterioration of glucose tolerance and a disproportionate increase in adiposity: 36% and 65% increase of relative weights of visceral and retroperitoneal adipose tissue, respectively, resulting effectively in a decrease of lean body mass. These results are consistent with the previously reported effects of sucrose feeding to SHR [33,34]. However, in a partly “mirror” congenic strain, SHR.1N (RGD ID: 628907), 12-week feeding of a high-fat diet resulted in a greater increase of body weight, adiposity, adipocyte size [23], and aggravation of glucose intolerance [17] compared to SHR while no nutrigenetic effects on lipid profile were found. These seemingly incongruous observations independently support the importance of the RNO20 region in the gene-environmental determination of MetS. The expected opposite effects did not manifest for several possible reasons. First, the transferred segment is twice as large in SHR.1N compared to BN.SHR20; second, a high-fat diet was used in all studies with SHR.1N, therefore nutrigenetic interactions distinct from those involving sucrose might be at play; third, the effects seen in congenic strain are not only due to the introgressed variants per se, but they also represent a product of gene–gene interactions with the alleles present in the genomic background of the recipient strain. This manifested e.g., in a triple-congenic strain BN-Lx.1K carrying a nearly identical segment of SHR RNO20 to that present in BN.SHR20 on the BN genomic background together with very small segments of SHR chromosome 4 including the mutant *Cd36* gene allele [10] and polydactylous strain chromosome 8 with mutant *Zbtb16* allele [35,36]). When fed a high-sucrose diet, this strain showed higher levels of triacylglycerols, non-esterified fatty acids, and worse glucose tolerance compared to BN and the single RNO4 and RNO8 congenic strains, yet showed the lowest adiposity, even compared to BN [16] (Supplementary Table S4). Several consomic strains were derived harboring the complete BN chromosome 20 on the genomic background of e.g., the Dahl Salt-Sensitive rat [37] or Sabra rat [38], but none of the studies addressed nutrigenetic effects on MetS. The isolation of the RNO20 region in the new congenic strain represents the first step on the way to fine mapping and identifying the causal genetic variation(s) responsible for the observed nutrigenetic interactions. Here, we prioritized in a first-pass the genes with variation in SHR coding regions together with evidence connecting them to MetS.

One of the genes we identified in this overlap is notch receptor 4 (*Notch4*), a crucial node in pathways of hepatic gluconeogenesis and lipogenesis [39]. In large genome-wide human association studies, variation in *Notch4* gene was associated with central obesity measures [40,41] and triacylglycerol levels [42]. Since Notch signalling was shown to suppress the expression of multiple metabolic genes integral to glycolysis or mitochondrial respiration [43], we might speculate that its malfunction due to the mutation in BN.SHR20 could contribute to the observed glucose intolerance and dyslipidemia. The small integral membrane protein 29 (*Smim29*), showing a predicted missense mutation in the SHR-derived differential segment in BN.SHR20, has been recently associated to several obesity-related indices [41], type 2 diabetes [44], fasting insulin [45], and HDL cholesterol [46]. However, there is very little information on the function of this gene beyond its original identification and positional cloning [47], making it an interesting candidate for further studies. Moreover, several of the identified genes showing sequence variation in untranslated regions belong to important metabolism regulators. For example, the glucagon-like peptide-1 receptor with 30 DNA variants in BN.SHR20 has been a heavily exploited target of type 2 diabetes and obesity therapies by its agonists [48], and the peroxisome proliferator-activated receptor beta/delta is crucial for fatty acid metabolism in the muscle [49]. The latter, like several other genes identified in this study (*Agpat1*, *Btnl3*,*5*,*8*, *Itpr3*, *Mapk14* or *Notch4*) connect the pathways relevant for metabolism with those for inflammation and oxidative stress, important players in metabolic syndrome pathogenesis [50].

We recognize several limitations of our study. First, only male rats were chosen for this study to maximize the homogeneity of the experimental groups on the genetic level in order to enable capturing subtle differences in metabolic and morphometric variables. Therefore, we could not address the potential sex-specific effects involved in the genetic architecture of MetS and its constituents [51]. Second, this study did not assess the microbiome, an crucial dynamic factor involved in processing external dietary cues and modulating the risk of MetS [52]. Furthermore, only short-term exposure to high-sucrose diet was utilized. While we cannot distinguish the effects attributable to the individual monosaccharides constituting sucrose, most of the detrimental metabolic effects are most likely attributable to fructose given its metabolic fate [53]. In addition, the first-pass prioritization focused on genes with variation in their coding regions combined with the prior evidence in rodent and human studies. It is clear that variants in non-coding regions and interactions of introgressed SHR alleles with specific variants within BN genomic background may be responsible for the observed phenotypic effects. Given the identification of significant gene-environmental interactions in the presented results, future targeted studies are warranted to comprehensively assess the role of the segment in the pathogenesis of MetS and determine the causal DNA variations. In summary, we established a new congenic model harboring a genomic region responsible for sensitizing towards sucrose-induced metabolic syndrome via nutrigenetic interactions.

## Figures and Tables

**Table 1:** Prioritization of candidate genes. Summary of genes carrying SHR-derived DNA variants in BN.SHR20 and, at the same time, showing significant associations to the constituents of metabolic syndrome in human genome-wide association studies (*p*-value ≤ 5.0 × 10^−8^). The complete list of variants is provided in Supplementary Table S2, the details on GWAS associations are available at https://www.ebi.ac.uk/gwas/genes/X (accessed on 7 July 2022), where X is the gene symbol.

BN.SHR20	Human GWAS				BN.SHR20	Human GWAS			
Gene with BN/SHR Variation	Glucose Tolerance	Obesity	Dyslipidemia	Blood Pressure	Gene with BN/SHR Variation	Glucose Tolerance	Obesity	Dyslipidemia	Blood Pressure
*Agpat1*	X			X	*Mapk14*		X		
*Anks1a*		X			*Mog*		X		
*Atp6v1g2*		X	X		*Mtch1*			X	
*Bag6*		X		X	*Mucl3*		X		
*Bak1*	X				*Ncr3*		X	X	X
*Brpf3*		X		X	*Nelfe*		X		
*Btbd9*	X	X			*Nfkbil1*	X	X		X
*Btnl3*	X		X		*Notch4*		X	X	
*Btnl8*			X		*Nudt3*	X	X	X	
*C2*		X	X		*Pacsin1*		X	X	
*Cdkn1a*			X		*Ppard*		X		X
*Col11a2*			X	X	*Ppt2*	X			
*Csnk2b*		X			*Prrc2a*		X		X
*Ddx39b*		X			*Rnf5*		X		
*Ehmt2*		X	X	X	*Rps10*		X		
*Fgd2*			X		*Scube3*		X		
*Fkbp5*		X			*Slc26a8*		X		
*Ggnbp1*	X	X		X	*Slc44a4*		X	X	X
*Glp1r*	X	X		X	*Smim29*	X	X	X	
*Grm4*		X		X	*Tap2*		X		X
*Hspa1b*		X			*Tapbp*		X		
*Ip6k3*		X	X		*Trim31*			X	
*Itpr3*	X	X	X		*Trim40*			X	
*Ltb*				X	*Tsbp1*	X	X	X	
*Ly6g5c*			X		*Vars1*	X	X		X
*Ly6g6c*				X	*Zfand3*	X			
*Mapk13*		X			*Zfp57*		X		

## Data Availability

The datasets generated during and/or analyzed during the current study are available in the Supplementary Materials and from the corresponding author upon a reasonable request.

## Supplementary Materials

The following are available online at https://www.mdpi.com/article/10.3390/nu14163428/s1, Table S1: Two-way ANOVA results for STRAIN and DIET as major factors and their interaction, Table S2: Summary of sequence variants between SHR and BN within the differential segment of BN.SHR20 congenic strain, Table S3: Non-synonymous amino-acid changes resulting from the SHR-derived variants in BN.SHR20., Table S4: Comparison of the metabolic effects of reciprocal introgression of genomic segments of chromosomes 4, 8, and 20 in congenic rat models.
