# Supplementary material for: Nutrigenetic Interaction of Spontaneously Hypertensive Rat Chromosome 20 Segment and High-Sucrose Diet Sensitizes to Metabolic Syndrome
Source: Nutrients. 2022 Aug 20;14(16):3428. doi: 10.3390/nu14163428 (PMC9416443; doi:10.3390/nu14163428)
Supplement: Supplementary file 1 [file nutrients-14-03428-s001.zip › Supplementary table S1.pdf]

Supplementary table S1. Two-way ANOVA results (p-values) for STRAIN and DIET as major factors and their interaction.

Trait	STRAIN	DIET	STRAIN*DIET INTERACTION
Body weight	0.119	**0.006**	0.31
Liver weight/100g	0.60	0.053	0.34
Heart weight/100g	0.63	**0.027**	0.09
Kidney weight	0.43	**9.5E-05**	**0.007**
Epididymal fat weight/100g	**0.0018**	**5.2E-06**	**0.015**
Retroperitoneal fat weight/100g	0.27	0.06	**0.027**
Triacylglycerols	**0.0006**	0.28	**0.0008**
Cholesterol	**4.8E-13**	**0.0015**	**0.013**
Glucose – 0 min	**7.4E-06**	0.59	0.83
Glucose – 30 min	**0.0003**	0.49	0.46
Glucose – 60 min	**0.0001**	0.23	0.29
Glucose – 120 min	**9.2E-09**	0.43	**0.021**
Glucose – 180 min	**2.2E-05**	0.42	0.30
AUC (OGTT)	**0.040**	0.49	0.21
Insulin	0.39	0.60	0.98
Free fatty acids	**5.15E-12**	0.058	**0.027**

Significant values are indicated in **bold**. AUC (OGTT)…area under the glycemic curve of the oral glucose tolerance test.
