# Supplementary material for: Nutrigenetic Interaction of Spontaneously Hypertensive Rat Chromosome 20 Segment and High-Sucrose Diet Sensitizes to Metabolic Syndrome
Source: Nutrients. 2022 Aug 20;14(16):3428. doi: 10.3390/nu14163428 (PMC9416443; doi:10.3390/nu14163428)
Supplement: Supplementary file 1 [file nutrients-14-03428-s001.zip › Supplementary Table S3.pdf]

Position on RNO 20 (bp)	Gene Symbol	Reference (BN) allele	SHR variant (BN.SHR20)	Reference Amino Acid	Variant Amino Acid
3077762	*Vars2*	T	A	Ser	Thr
3691116	*Apom*	T	C	Met	Thr
4173424	*Notch4*	A	G	Val	Ala
4270058	*Btnl8*	T	A	Ile	Asn
4304918	*Btnl7*	C	G	His	Gln
4308932	*Btnl7*	T	C	Trp	Arg
4366798	*Btnl5*	C	G	Pro	Ala
4366804	*Btnl5*	G	C	Val	Leu
4366907	*Btnl5*	G	A	Ala	Thr
4366948	*Btnl5*	G	T	Glu	Asp
4366959	*Btnl5*	C	G	Pro	Arg
4368527	*Btnl5*	G	A	Glu	Lys
4368528	*Btnl5*	A	T	Glu	Val
4368540	*Btnl5*	G	A	Gly	Glu
4368544	*Btnl5*	A	G	Ile	Met
4368545	*Btnl5*	C	T	His	Tyr
4824317	*Slc39a7*	G	C	Asp	His
4824834	*Slc39a7*	C	T	Thr	Met
5619004	*Smim29*	G	C	His	Asp
7767505	*Cmtr1*	T	C	Gln	Arg

Supplementary Table S3. Summary of nonsynonymous aminoacid changes resulting from the SHR-derived variants in BN.SHR20 (excluding the Rt1 and olfactory receptor genes). The complete list of variants is provided in Supplementary Table S2. The genomic position of the variants is given in basepairs (bp) of the rat chromosome 20 (RNO20) according to NCBI Rattus norvegicus Annotation Release 108, Rattus norvegicus mRatBN7.2 (GCF_015227675.2 assembly).
