# Supplementary material for: Nutrigenetic Interaction of Spontaneously Hypertensive Rat Chromosome 20 Segment and High-Sucrose Diet Sensitizes to Metabolic Syndrome
Source: Nutrients. 2022 Aug 20;14(16):3428. doi: 10.3390/nu14163428 (PMC9416443; doi:10.3390/nu14163428)
Supplement: Supplementary file 1 [file nutrients-14-03428-s001.zip › Supplementary table S4.pdf]

Strain	SHR-derived segment			vs. BN					
	RNO4	RNO8	RNO20	Adiposity	Glucose intolerance	Triacylglycerols	Cholesterol	Free fatty acids	References
BN.SHR4				↑	↑	↑	–	↑	[1]
BN-*Lx*				↓	↑	↑	–	↑	[1]
BN.SHR20				↑	↑	↑	↑	↑	current study
BN-*Lx*.SHR4.SHR20				↓	↑	↑	–	↑	[1]
	BN-derived segment			vs. SHR					
SHR.BN-RT1^{N}				↑	↑	–	–	–	[2]
SHR-*Lx*				↓	↓	↑	–	–	[3],[4]
SHR.BN4				↓	↓	↓	–	↓	[5]

Supplementary Table S4. Comparison of the metabolic effects of reciprocal introgression of genomic segments of chromosomes 4, 8, and 20 in congenic rat models.

1. Seda, O.; Sedova, L.; Kazdova, L.; Krenova, D.; Kren, V. Metabolic characterization of insulin resistance syndrome feature loci in three brown Norway-derived congenic strains. *Folia Biol (Praha)* **2002**, *48*, 81-88.
2. Pausova, Z.; Sedova, L.; Berube, J.; Hamet, P.; Tremblay, J.; Dumont, M.; Gaudet, D.; Pravenec, M.; Kren, V.; Kunes, J. Segment of rat chromosome 20 regulates diet-induced augmentations in adiposity, glucose intolerance, and blood pressure. *Hypertension* **2003**, *41*, 1047-1055, doi:10.1161/01.HYP.0000064347.49341.0B.
3. Seda, O. Comparative gene map of hypertriglyceridaemia. *Folia Biol (Praha)* **2004**, *50*, 43-57.
4. Seda, O.; Liska, F.; Sedova, L.; Kazdova, L.; Krenova, D.; Kren, V. A 14-gene region of rat chromosome 8 in SHR-derived polydactylous congenic substrain affects muscle-specific insulin resistance, dyslipidaemia and visceral adiposity. *Folia Biol (Praha)* **2005**, *51*, 53-61.
5. Pravenec, M.; Zidek, V.; Simakova, M.; Kren, V.; Krenova, D.; Horky, K.; Jachymova, M.; Mikova, B.; Kazdova, L.; Aitman, T.J., et al. Genetics of Cd36 and the clustering of multiple cardiovascular risk factors in spontaneous hypertension. *J Clin Invest* **1999**, *103*, 1651-1657, doi:10.1172/JCI6691.
